# Supplementary material for: Computational simulation of the reactive oxygen species and redox network in the regulation of chloroplast metabolism
Source: PLoS Comput Biol. 2020 Jan 17;16(1):e1007102. doi: 10.1371/journal.pcbi.1007102 (PMC6992225; doi:10.1371/journal.pcbi.1007102)
Supplement: S1 Text — (PDF) [file pcbi.1007102.s001.pdf]

S1 Text

A Model equation

To represent the thiol-disulfide redox network of the cell three different models were constructed. The FTR model (Fig. 1A), the FNR model (Fig. 2A) and after their merging the FTR-FNR model (Fig. 3A).

A1 FTR network model

In the model represented in Fig. 1A. FD and H_2O_2 are external quantities and their concentration is constant. The variables FTR, TRX-f1, FBPase and 2CysPRX exhibit a reduced and oxidized form (reaction equation Suppl Table 7). The rate expressions are read

$$v_1 = k_1 * ([FDX_{red}] * [FDX_{red}] * [FTR_{ox}] - \frac{[FDX_{ox}]*[FDX_{ox}]*[FTR_{red}]}{K_{eq\_FdFTR}}) \quad (A.1)$$

$$v_2 = k_2 * ([FTR_{red}] * [Trxf1_{ox}] - \frac{[FTR_{ox}]*[Trxf1_{red}]}{K_{eq\_FTRTrxf1}}) \quad (A.2)$$

$$v_3 = k_3 * ([Trxf1_{red}] * [FBPase_{ox}] - \frac{[Trxf1_{ox}]*[FBPase_{red}]}{K_{eq\_Trxf1FBPase}}) \quad (A.3)$$

$$v_4 = k_4 * ([Trxf1_{red}] * [2CysPrx_{ox}] - \frac{[Trxf1_{ox}]*[2CysPrx_{red}]}{K_{eq\_Trxf12CP}}) \quad (A.4)$$

$$v_5 = k_5 * [2CysPrx_{red}] * [H_2O_2] \quad (A.5)$$

FD is implemented with a constant redox state of 50 % reduced and oxidized. Constant H_2O_2 concentration vary from 0 to 10 µM in different simulation.

Concerning the two states (oxidized and reduced) of each component, the conserved quantities are:

FTR pool:	$FTR_{red} + FTR_{ox} = FTR_{total}$	(A.6)
TRX-f1 pool:	$TRX\text{-}f1_{red} + TRX\text{-}f1_{ox} = TRX\text{-}f1_{total}$	(A.7)
FBPase pool:	$FBPase_{red} + FBPase_{ox} = FBPase_{total}$	(A.8)
2CysPRX pool:	$2CysPRX_{red} + 2CysPRX_{ox} = 2CysPRX_{total}$	(A.9)

The differential equations of the independent variables FTR_{red}, $TRX\text{-}f1_{red}$, $FBPase_{red}$, $2CysPRX_{red}$ used for simulating FTR network model are:

$$\frac{d[FTR_{red}]}{dt} = +v_1 - v_2 \quad (A.10)$$

$$\frac{d[Trxf1_{red}]}{dt} = +v_2 - v_3 - v_4 \quad (A.11)$$

$\frac{d[FBPase_{red}]}{dt} = +v_3$ (A.12)

$\frac{d[2CysPrx_{red}]}{dt} = +v_4 - v_5$ (A.13)

A2 FNR network model

The FNR network model (Fig 3A) consists of FD, FNR, NADPH, NTRC, 2CysPRX and H_2O_2. Each component has an oxidized and reduced form. Only FNR exhibits three forms; reduced, half reduced and oxidized. In order to mimic metabolic NADPH consumption an estimated rate of NADPH decrease was included (v11). Each reaction (except v5 and v11) is reversible. The equilibrium constants are calculated from redox potential of involved components (material and methods). The rate expressions of FNR network model are

$v_5 = k_5 * [2CysPrx_{red}] * [H_2O_2]$ (A.14)

$v_6 = k_{+6} * [FDX_{red}] * [FNR_{ox}] - k_{-6} * [FDX_{ox}] * [FNR_{semired}]$ (A.15)

$v_7 = k_{+7} * [FDX_{red}] * [FNR_{semired}] - k_{-7} * [FDX_{ox}] * [FNR_{red}]$ (A.16)

$v_8 = k_{+8} * [FNR_{red}] * [NADP^+] - k_{-8} * [FNR_{ox}] * [NADPH]$ (A.17)

$v_9 = k_9 * ([NADPH] * [NTRC_{ox}] - \frac{[NADP^+]*[NTRC_{red}]}{K_{eq\_NADPHNTRC}})$ (A.18)

$v_{10} = k_{10} * ([NTRC_{red}] * [2CysPrx_{ox}] - \frac{[NTRC_{ox}]*[2CysPrx_{red}]}{K_{eq\_NTRC2CP}})$ (A.19)

$v_{11} = 2.05243e3$ (A.20)

FD and H_2O_2 concentrations are considered to be constants. FD is constantly reduced at 50 % and the H_2O_2 concentration varies from 0 to 100 µM in different simulations.

The equations of conserved variables are:

NADPH/$NADP^+$ pool: NADPH + $NADP^+$ = NADPH/$NADP^+_{total}$ (A.21

NTRC pool: $NTRC_{red}$ + $NTRC_{ox}$ = $NTRC_{total}$ (A.22)

2CysPRX pool: $2CysPRX_{red}$ + $2CysPRX_{ox}$ = $2CysPRX_{total}$ (A.23)

The differential equation of the variables used for simulating FNR network model are:

$\frac{d[FNR_{ox}]}{dt} = -v_6 + v_8$ (A.24)

$\frac{d[FNR_{semired}]}{dt} = +v_6 - v_7$ (A.25)

$\frac{d[FNR_{red}]}{dt} = +v_6 - v_8$ (A.26)

$\frac{d[NADPH]}{dt} = +v_8 - v_9 - v_{11}$ (A.27)

$$\frac{d[NTRC_{red}]}{dt} = +v_9 - v_{10} \tag{A.28}$$

$$\frac{d[2CysPrx_{red}]}{dt} = +v_{10} - v_5 \tag{A.29}$$

A3 FTR-FNR network model

The FTR-FNR network model (Fig. 5A) combines both submodels. FD and H_2O_2 are considered to be constant quantities. The redox state of FD is set constant to 50 % reduced form and the concentration of H_2O_2 varies from 0 nM to 100 µM. The variables are FTR, TRX-f1, FBPase, 2CysPRX, FNR, NADPH/NADP$^+$ couple and NTRC. Each variable (except FNR) is represented in an oxidized and reduced state. FNR is implemented in three forms, oxidized, reduced and half reduced. Each reaction (except the metabolic consumption of NADPH; v11 and H_2O_2 detoxification; v5) is reversible. The equilibrium constants are calculated from redox potentials of involved components (see Material and Methods). The rate expressions of FTR-FNR network model are

$$v_1 = k_1 * ([FDX_{red}] * [FDX_{red}] * [FTR_{ox}] - \frac{[FDX_{ox}]*[FDX_{ox}]*[FTR_{red}]}{K_{eq\_FdFTR}}) \tag{A.30}$$

$$v_2 = k_2 * ([FTR_{red}] * [Trxf1_{ox}] - \frac{[FTR_{ox}]*[Trxf1_{red}]}{K_{eq\_FTRTrxf1}}) \tag{A.31}$$

$$v_3 = k_3 * ([Trxf1_{red}] * [FBPase_{ox}] - \frac{[Trxf1_{ox}]*[FBPase_{red}]}{K_{eq\_Trxf1FBPase}}) \tag{A.32}$$

$$v_4 = k_4 * ([Trxf1_{red}] * [2CysPrx_{ox}] - \frac{[Trxf1_{ox}]*[2CysPrx_{red}]}{K_{eq\_Trxf12CP}}) \tag{A.33}$$

$$v_5 = k_5 * [2CysPrx_{red}] * [H_2O_2] \tag{A.34}$$

$$v_6 = k_{+6} * [FDX_{red}] * [FNR_{ox}] - k_{-6} * [FDX_{ox}] * [FNR_{semired}] \tag{A.35}$$

$$v_7 = k_{+7} * [FDX_{red}] * [FNR_{semired}] - k_{-7} * [FDX_{ox}] * [FNR_{red}] \tag{A.36}$$

$$v_8 = k_{+8} * [FNR_{red}] * [NADP^+] - k_{-8} * [FNR_{ox}] * [NADPH] \tag{A.37}$$

$$v_9 = k_9 * ([NADPH] * [NTRC_{ox}] - \frac{[NADP^+]*[NTRC_{red}]}{K_{eq\_NADPHNTRC}}) \tag{A.38}$$

$$v_{10} = k_{10} * ([NTRC_{red}] * [2CysPrx_{ox}] - \frac{[NTRC_{ox}]*[2CysPrx_{red}]}{K_{eq\_NTRC2CP}}) \tag{A.39}$$

$$v_{11} = 2.05243e3 \tag{A.40}$$

The conserved quantities of the variables are:

FTR pool: $FTR_{red} + FTR_{ox} = FTR_{total}$ (A.41)

TRX-f1 pool: $TRX\text{-}f1_{red} + TRX\text{-}f1_{ox} = TRX\text{-}f1_{total}$ (A.42)

FBPase pool: $FBPase_{red} + FBPase_{ox} = FBPase_{total}$ (A.43)

2CysPRX pool: $2CysPRX_{red} + 2CysPRX_{ox} = 2CysPRX_{total}$ (A.44)

$NADPH/NADP^{+}$ pool: $NADPH + NADP^{+} = NADPH/NADP^{+}_{total}$ (A.45)

NTRC pool: $NTRC_{red} + NTRC_{ox} = NTRC_{total}$ (A.46)

The complete differential equation system introduced into MATLAB is the following:

$$\frac{d[FTR_{red}]}{dt} = +v_1 - v_2 \quad (A.47)$$

$$\frac{d[Trxf1_{red}]}{dt} = +v_2 - v_3 - v_4 \quad (A.48)$$

$$\frac{d[FBPase_{red}]}{dt} = +v_3 \quad (A.49)$$

$$\frac{d[2CysPrx_{red}]}{dt} = +v_4 - v_5 + v_{10} \quad (A.50)$$

$$\frac{d[FNR_{ox}]}{dt} = -v_6 + v_8 \quad (A.51)$$

$$\frac{d[FNR_{semired}]}{dt} = +v_6 - v_7 \quad (A.52)$$

$$\frac{d[FNR_{red}]}{dt} = +v_6 - v_8 \quad (A.53)$$

$$\frac{d[NADPH]}{dt} = +v_8 - v_9 - v_{11} \quad (A.54)$$

$$\frac{d[NTRC_{red}]}{dt} = +v_9 - v_{10} \quad (A.55)$$

B Choice of parameter

The parameters of the network models are given in Table B.1. Most of the parameters are available from literature. The units of concentrations are µM/s. The rate constants are second or third order. Unknown rate constants are fitted (see Material and Methods). The physiological concentrations of network components are calculated for 1 µg Chl and 66 µL stromal volume. If needed, the concentrations of isoforms are summed up.

Table B1: Parameter list

parameter	value	Reference / comment
k_{+1}	$2.307861608796e^{+2}\ \mu M^{-1}\mu M^{-1}s^{-1}$	fitted
k_{+2}	$3.0857906493e^{-2}\ \mu M^{-1}s^{-1}$	fitted
k_{+3}	$2.9616e^{-2}\ \mu M^{-1}s^{-1}$	Calculated from Collin *et al.* [1]
k_{+4}	$1.84e^{-3}\ \mu M^{-1}s^{-1}$	Collin *et al.* [1]
k_{+5}	$5.1e^{-1}\ \mu M^{-1}s^{-1}$	Parsonage *et al.* [2]
k_{+6}	$4.1e^{2}\ \mu M^{-1}s^{-1}$	Cassan *et al.* [3]
k_{-6}	$1.5e^{2}\ \mu M^{-1}s^{-1}$	Cassan *et al.* [3]
k_{+7}	$4.1e^{2}\ \mu M^{-1}s^{-1}$	Cassan *et al.* [3]
k_{-7}	$2.0e^{1}\ \mu M^{-1}s^{-1}$	Cassan *et al.* [3]
k_{+8}	$1.83e^{1}\ \mu M^{-1}s^{-1}$	Aliverti *et al.*[4]
k_{-8}	$1.04617638957139e^{2}\ \mu M^{-1}s^{-1}$	fitted
k_{+9}	$1.489e^{-1}\ \mu M^{-1}s^{-1}$	Dai *et al.* [5]
k_{+10}	$2.1e^{-1}\ \mu M^{-1}s^{-1}$	Pérez-Ruiz *et al.* [6]
k_{+11}	2.05243e3	estimated
$Keq\_{}_{FdFTR}$	$9.22e^{2}$	calculated
$Keq\_{}_{FTRTRX\text{-}f1}$	$1.525e^{1}$	calculated
$Keq\_{}_{TRX\text{-}f1FBPase}$	$5.697\ e\text{-}^{1}$	calculated
$Keq\_{}_{TRX\text{-}f12CP}$	$3.856\ e\text{-}^{1}$	calculated
$Keq\_{}_{NADPHNTRC}$	$1.6278e^{4}$	calculated
$Keq\_{}_{NTRC2CP}$	$6.558e\text{-}^{3}$	calculated
FD_{total}	69 µM	calculated from Hall *et al.* [7]
FD_{red_fix}	34.5 µM	Estimated (50 % reduced)
FTR_{total}	4.7727 µM	calculated from Yoshida and Hisabori [8]
$TRX\text{-}f1_{total}$	1.899 µM	Calculated from König *et al.* [9]
$FBPase_{total}$	7.13267 µM	calculated from Peltier *et al.* [10]
$2CysPRX_{total}$	63.3 µM	Calculated from Peltier *et al.* [10]
FNR_{total}	4.361 µM	Calculated from Peltier *et al.* [10]
$NADPH_{total}$	100 µM	Heber and Santarius [11]
$NTRC_{total}$	3.165 µM	Calculated from König *et al.* [9]
$H_2O_2\_{}_{total}$	0 - 100 µM	Estimated

Appendix C Fitting results

Data of FTR-dependent reduction of TRX-f1 are taken from Yoshida and Hisabori [33]. For fitting of unknown parameters, a model was developed that includes the components of

measurements (S4 Figs 4 and 5). The kinetic parameters are fitted by different optimization tools in MATLAB.

Output parameters of the global GA-optimization vs. the local lsqnonlin and fminunc tools. For the data set TRX-f1 the parameters are given for three local minima. The values of fval represent the measures of quality of the optimization. The error margins for the output parameters of the GA-optimization were calculated from differencies between GA and local algorithms output parameters. For a comparison, we applied the two MATLAB local algorithms: “fminunc” and “lsqnonlin”. The tool “fminunc” is the Matlab’s standard optimizer, “lsqnonlin” solves nonlinear data-fitting problems. All the optimization algorithms require the user to specify an initial guess for the parameters. Here we used the output parameters of the GA-optimization at different local minima as the initial guess. All the final parameters of the paper are results of the global and local optimization.

Table C1: Output parameter list of GA-optimization vs. local optimization.

Data Set	Parameter, $\mu M^{-1}\ s^{-1}$	GA	lsqnonlin	Fminunc
TRX-f1, min. 1	k+1	$(2.308 \pm 0.001)\cdot 10^{2}$	$2.308\cdot 10^{2}$	$2.308\cdot 10^{2}$
	k+2	$(2.1 \pm 0.9)\cdot 10^{-2}$	$3.086\cdot 10^{2}$	$3.086\cdot 10^{2}$
	k-8	$(1.046 \pm 0.001)\cdot 10^{2}$	$1.046\cdot 10^{2}$	$1.046\cdot 10^{2}$
	fval	0.1620	0.0633	0.0633
TRX-f1, min. 2	k+1	$(1.8 \pm 0.1)\cdot 10^{-1}$	$1.728\cdot 10^{-1}$	$1.728\cdot 10^{-1}$
	k+2	$(2.2 \pm 1.7)\cdot 10^{-1}$	$5.084\cdot 10^{-2}$	$5.084\cdot 10^{-2}$
	k-8	1.9 ± 1.6	$3.646\cdot 10^{-1}$	$3.646\cdot 10^{-1}$
	fval	0.4276	0.0199	0.0199
TRX-f1 min. 3	k+1	$(1.4 \pm 2.0)\cdot 10^{-2}$	$4.553\cdot 10^{-2}$	$8.079\cdot 10^{-2}$
	k+2	$(1.5 \pm 1.4)\cdot 10^{-1}$	$5.818\cdot 10^{-2}$	$5.506\cdot 10^{-2}$
	k-8	$(1.723 \pm 0.001)\cdot 10^{2}$	$1.723\cdot 10^{2}$	$1.894\cdot 10^{2}$
	fval	0.1624	0.0333	0.0234
Trxf2	k+1	$(4.033 \pm 0.001)\cdot 10^{2}$	$4.033\cdot 10^{2}$	$4.033\cdot 10^{2}$
	k_Trxf2	$(3.28 \pm 0.3)\cdot 10^{-2}$	$3.530\cdot 10^{-2}$	$3.530\cdot 10^{-2}$
	k-8	$(1.353 \pm 0.001)\cdot 10^{2}$	$1.353\cdot 10^{2}$	$1.353\cdot 10^{2}$
	fval	0.0747	0.0671	0.0671
Trxm1	k+1	$(5.269 \pm 0.001)\cdot 10^{2}$	$5.269\cdot 10^{2}$	$5.269\cdot 10^{2}$
	k_Trxm1	$(3.9 \pm 0.9)\cdot 10^{-3}$	$4.882\cdot 10^{-3}$	$4.882\cdot 10^{-3}$
	k-8	$(2.035 \pm 0.001)\cdot 10^{2}$	$2.035\cdot 10^{2}$	$2.035\cdot 10^{2}$
	fval	0.0918	0.0175	0.0175

D Redox potentials of network components.

The redox potentials of TRX-f1, FTR, 2CysPRX, NTRC and NADPH are calculated at each time step by Nernst equation at room temperature.

Table D1: Redoxpotentials

component	Em [mV], pH = 7	Reference / comment	E [mV], t = 0
TRX-f1	- 291,3	Collin *et al.* (2003) [7]	-368,82
FTR	- 326,3	Schürmann *et al.* (2000) [12]	-344,12
FD	- 414	Buckel *et al.* (2013) [13]	/
2CysPRX	- 310	Yoshida & Hisabori (2017) [8]	-302,04
FBPase	- 305	Schürmann *et al.* (2000) [12]	-322,82
NTRC	- 245,43	Yoshida & Hisabori (2017) [8]	-263,26
NADPH	- 370	Huang et al. (2012) [14]	-370
FNR	- 344	Pueyo *et al.* (1991) [15]	/

REFERENCES

1. Collin V, Issakidis-Bourguet E, Marchand C, Hirasawa M, Lancelin JM, Knaff DB, Miginiac-Maslow M. The Arabidopsis plastidial thioredoxins: new functions and new insights into specificity. Journal of Biological Chemistry. 2003; 278: 23747-23752.ligomeric State of AhpC, a bacterial Peroxiredoxin, Biochemistry. 2005; 44: 10583-10592
2. Parsonage D, Youngblood DS, Sarma GN, Wood ZA, Karplus PA, Poole LB. Analysis of the Link between Enzymatic Activity and Oligomeric State of AhpC, a bacterial Peroxiredoxin, Biochemistry. 2005; 44: 10583-10592
3. Cassan N, Lagoutte B, Setif P. Ferredoxin-NADPH+ Reductase; Kinetics of electron transfer, transient intermediates and catalytic actitrvities studied by flash-absorption spectroskopiy with isolated photosystem I and ferredoxin, The Journal of Biological chemistry. 2005; 280: 25960- 25972.
4. Aliverti A, Pandini V, Zanetti G. Domain exchange between isoforms of ferredoxin-NADP+ reductase produces a functional enzyme: Biochim. Biophys. Acta. 2004; 1696: 93-101.
5. Dai C, Wang MH. Isolation and characterization of thioredoxin and NADPH-dependent thioredoxin reductase from tomato (Solanum lycopersicum), BMP Rep. 2011; 44: 692-697.
6. Pèrez-Ruiz JM, Spinola MC, Kirchsteiger K, Moreno J, Sahrawy M, Cejudo FJ. Rice NTRC is a high-efficiency redox system for chloroplasts protection against oxidative damage, The Plant Cell. 2006; 18: 2356-2368.

7. Hall DO, Rao KK. Ferredoxin, photosynthesis I, photoynthetic electron transport and photophosphorylation, In: Encyclopedia of Plant Physiology, Trebst A and Avron M, eds. 1977; 5: 206-216.
8. Yoshida K, Hisabori T. Distinct electron transfer from ferredoxin-thioredoxin reductase to multiple thioredoxin isoforms in chloroplast. Biochemical Journal. 2017; 474: 1347-1360.
9. König J, Muthuramalingam M, Dietz KJ. Mechanisms and dynamics in the thiol/disulfide redox regulatory network: transmitters, sensors and targets. Current Opinion in Plant Biology. 2012; 15: 261-268.
10. Peltier JB, Cai Y, Sun Q, Zabrouskov V, Giacomelli L, Rudella A, Ytterberg AJ, Rutschow H, and van Wijk KJ. () The Oligomeric Stromal Proteome of Arabidopsis thaliana Chloroplasts. Molecular & Cellular Proteomics. 2006; 5.1: 114-133.
11. Heber U, Santarius KA. Compartmentation and reduction of pyridine nucleotides in relation to photosynthesis. Biochim Biophys Acta. 1965; 109: 390-408.
12. Schürmann P, Jacquot JP. Plant Thioredoxin System Revisited. Annu. Rev. Plant Physiol. Plant Mol. Biol. 2000; 51: 371-400.
13. Buckel W, Thauer RK. Energy conservation via electron bifurcating ferredoxin reduction and proton Na^+ translocating ferredoxin oxidation. Biochimica et Biophysica Acta – Bioenergetics. 2013; 1827: 94-113.
14. Huang FC, Sung PH, Do YY, Huang PL. Differential expression and functional characterization of the NADPH cytochrome P450 reductase genes from *Nothapodytes foetida*. Plant Sci. 2012; 190: 16-23.
15. Pueyo JJ, Gomez-Moreno C, Mayhew SG. Oxidation-reduction potentials of ferredoxin-$NADP^+$ reductase and flavodoxin from Anabena PCC 7119 and their electrostatic and covalent complexes. Eur J Biochem. 1991; 202: 1065-1071.
