# Supplementary figures and images for: Computational simulation of the reactive oxygen species and redox network in the regulation of chloroplast metabolism
Source: PLoS Comput Biol. 2020 Jan 17;16(1):e1007102. doi: 10.1371/journal.pcbi.1007102 (PMC6992225; doi:10.1371/journal.pcbi.1007102)

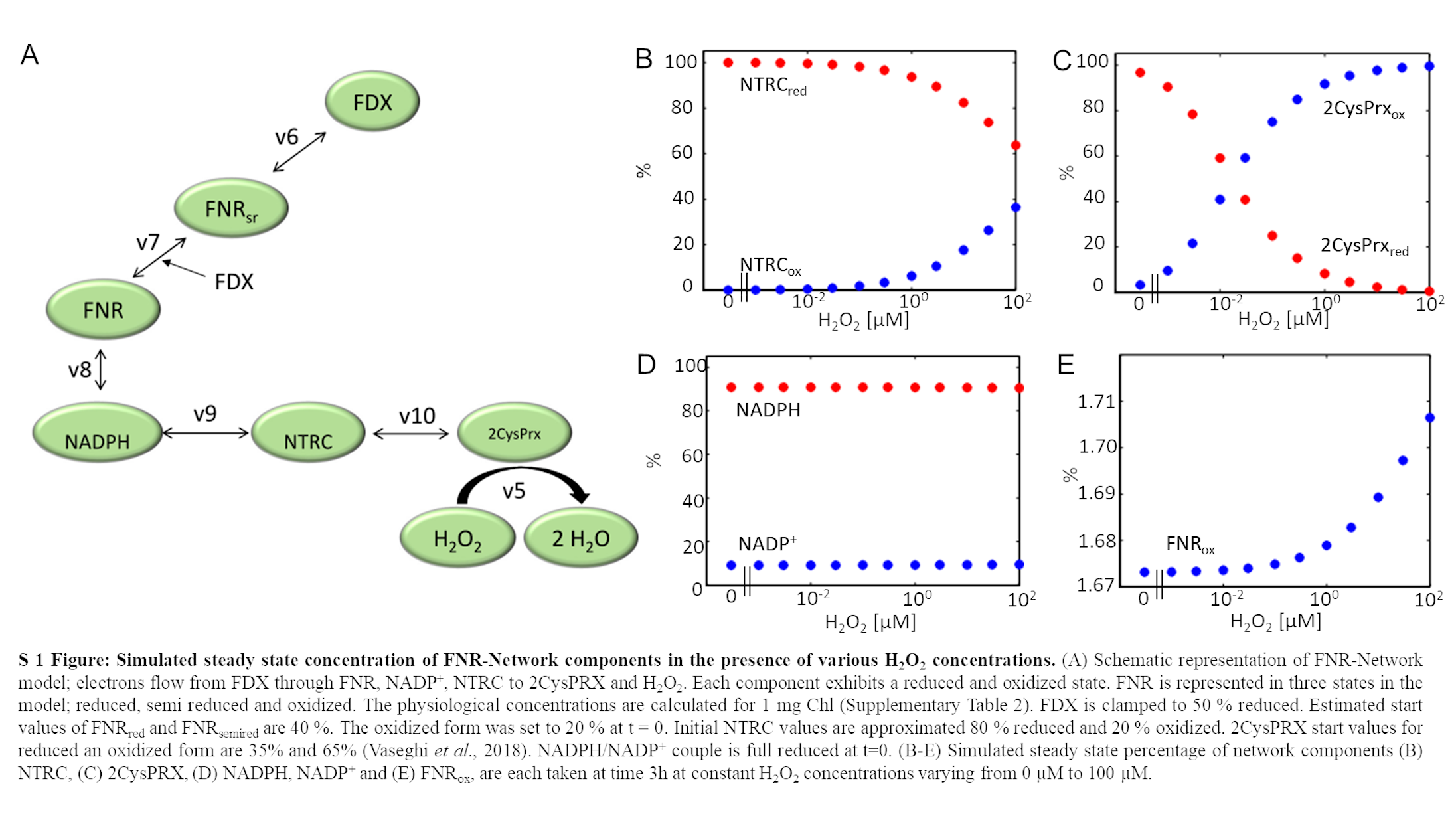

Supplement: S1 Fig — (TIFF) [file pcbi.1007102.s002.tiff]

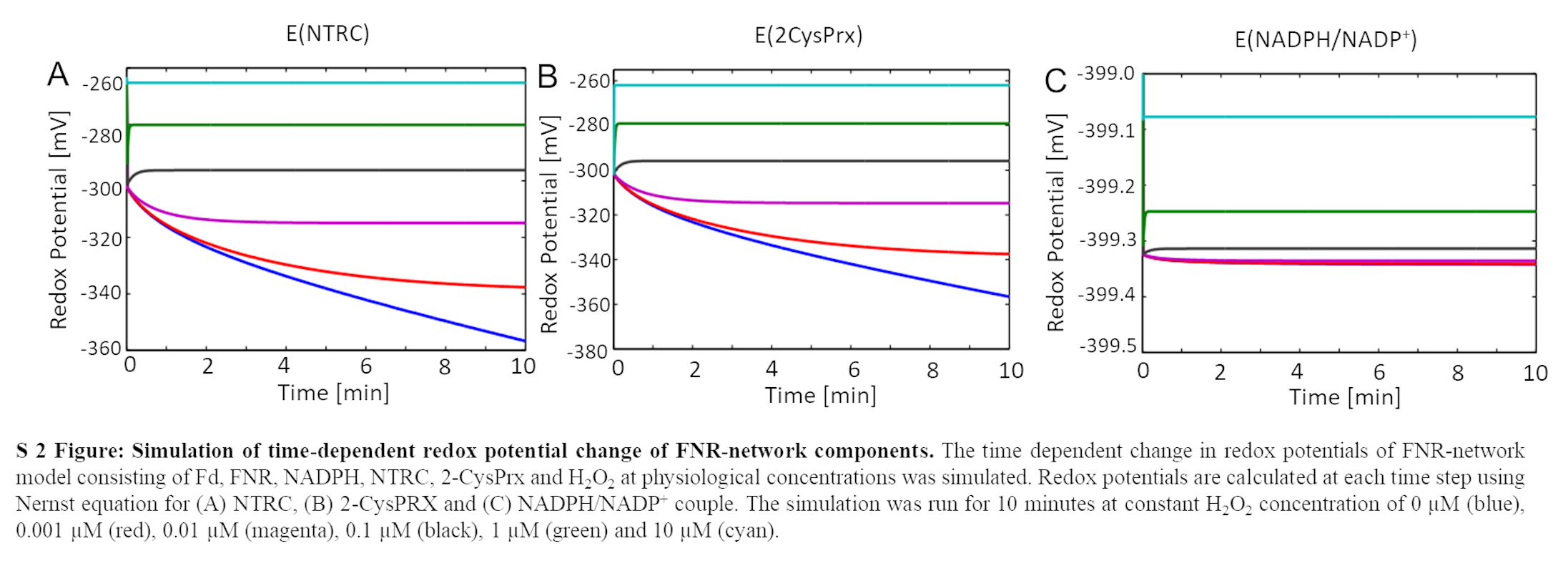

Supplement: S2 Fig — (TIFF) [file pcbi.1007102.s003.tiff]

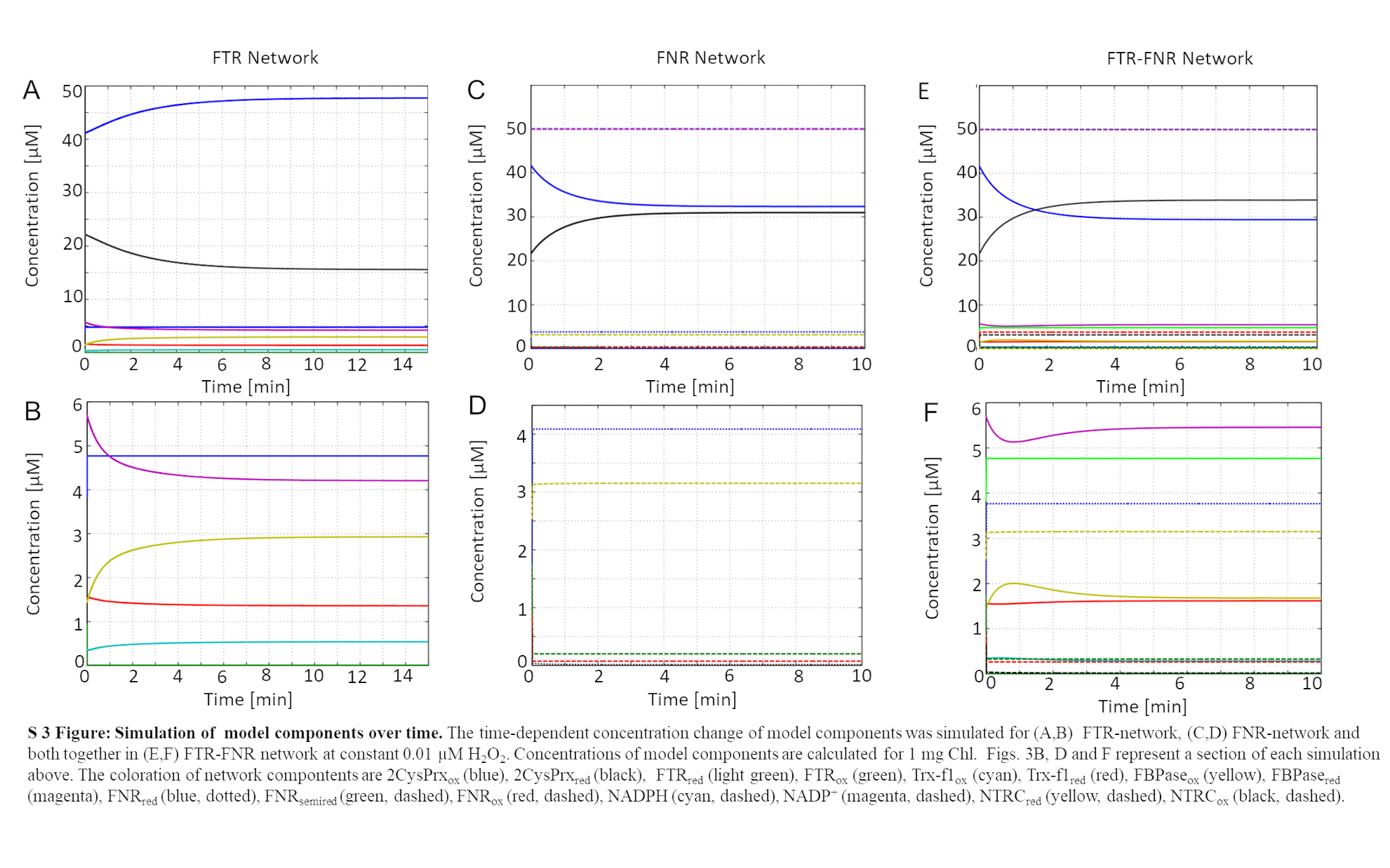

Supplement: S3 Fig — (TIFF) [file pcbi.1007102.s004.tiff]

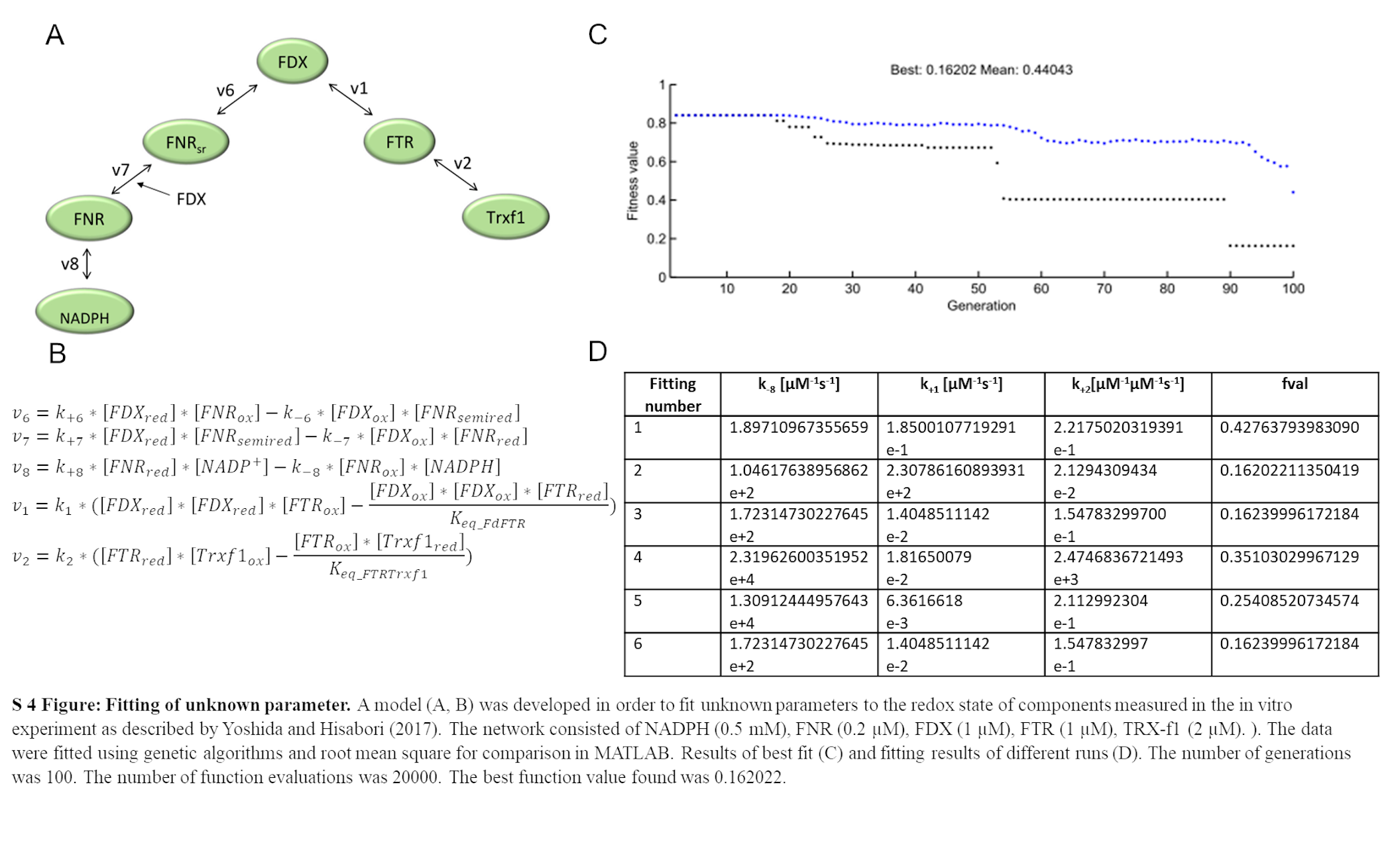

Supplement: S4 Fig — (TIFF) [file pcbi.1007102.s005.tiff]

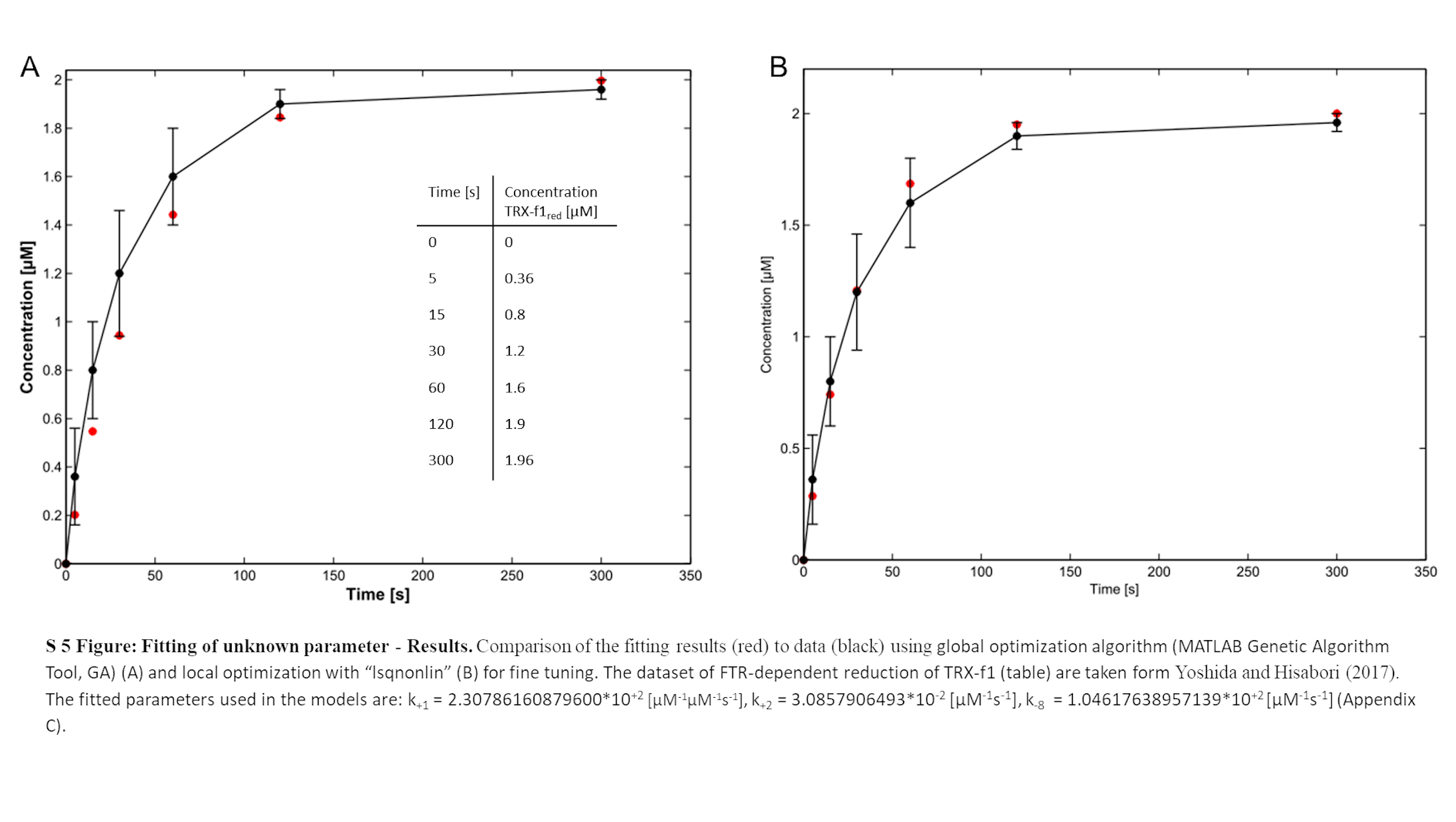

Supplement: S5 Fig — (TIFF) [file pcbi.1007102.s006.tiff]

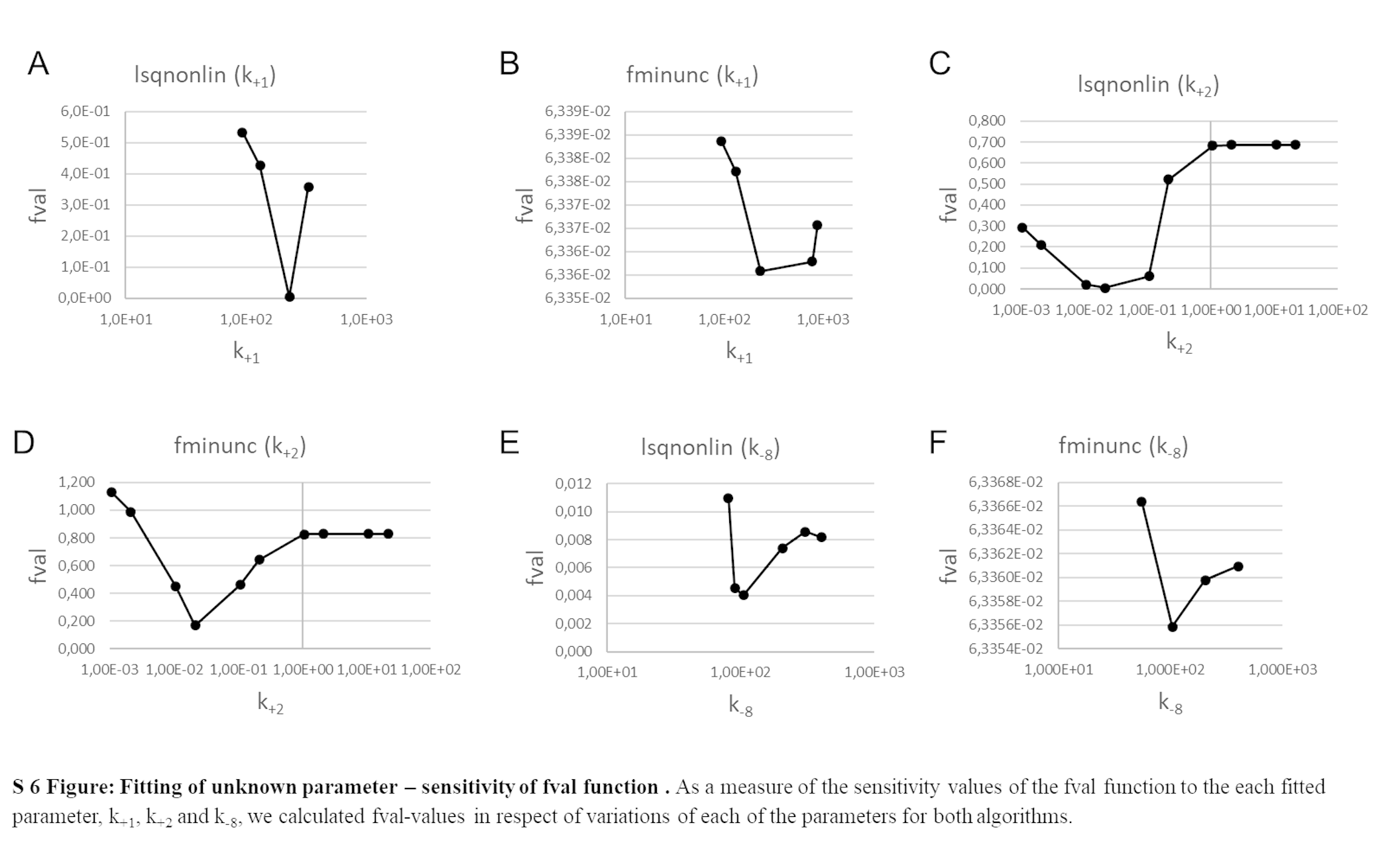

Supplement: S6 Fig — (TIFF) [file pcbi.1007102.s007.tiff]

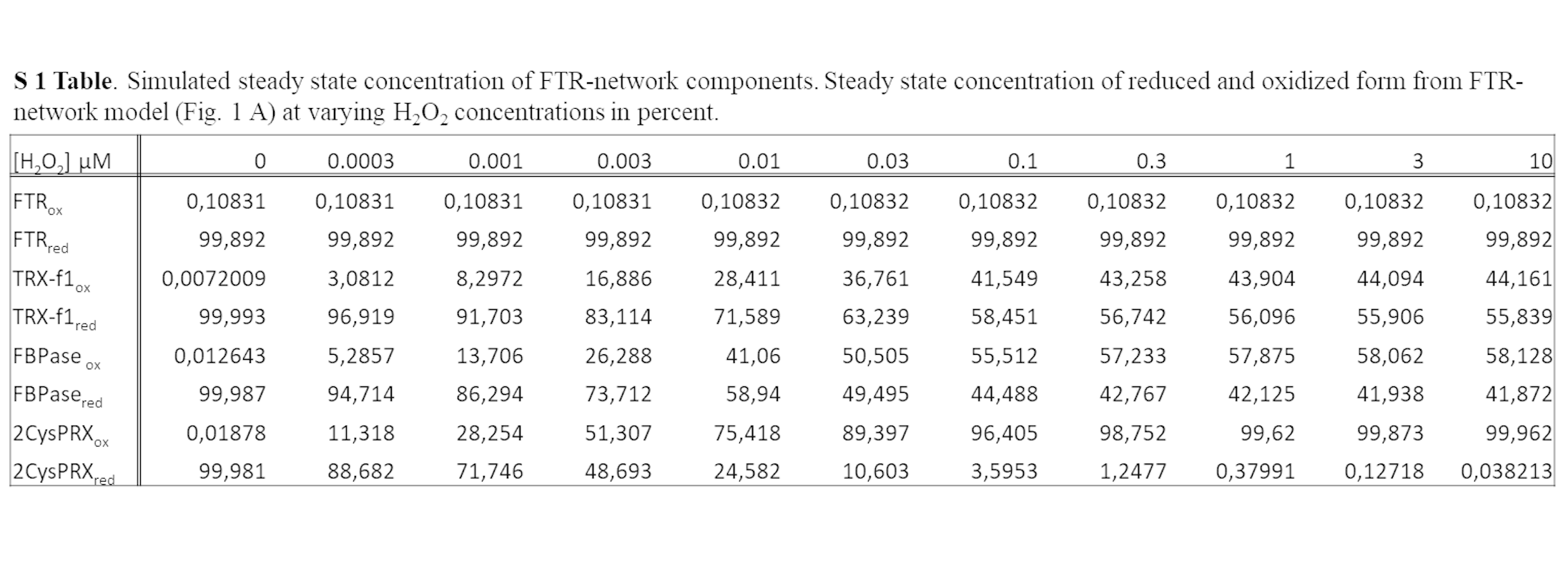

Supplement: S1 Table — (TIFF) [file pcbi.1007102.s008.tiff]

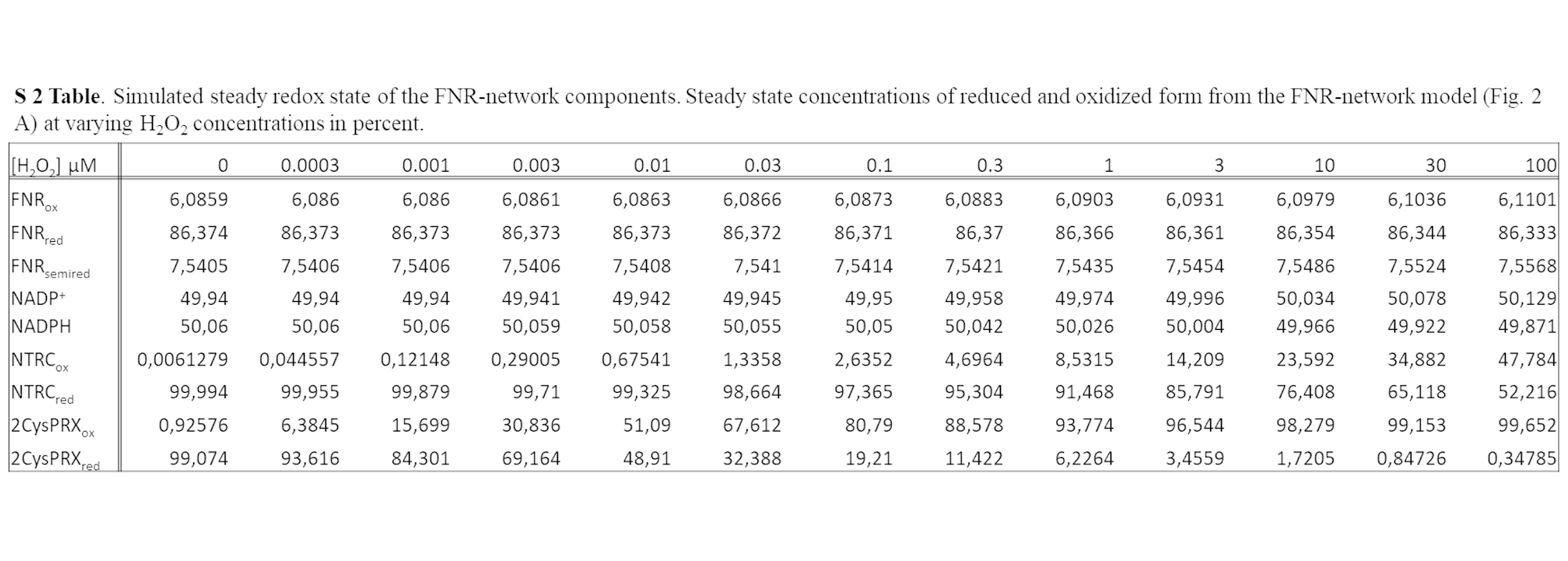

Supplement: S2 Table — (TIFF) [file pcbi.1007102.s009.tiff]

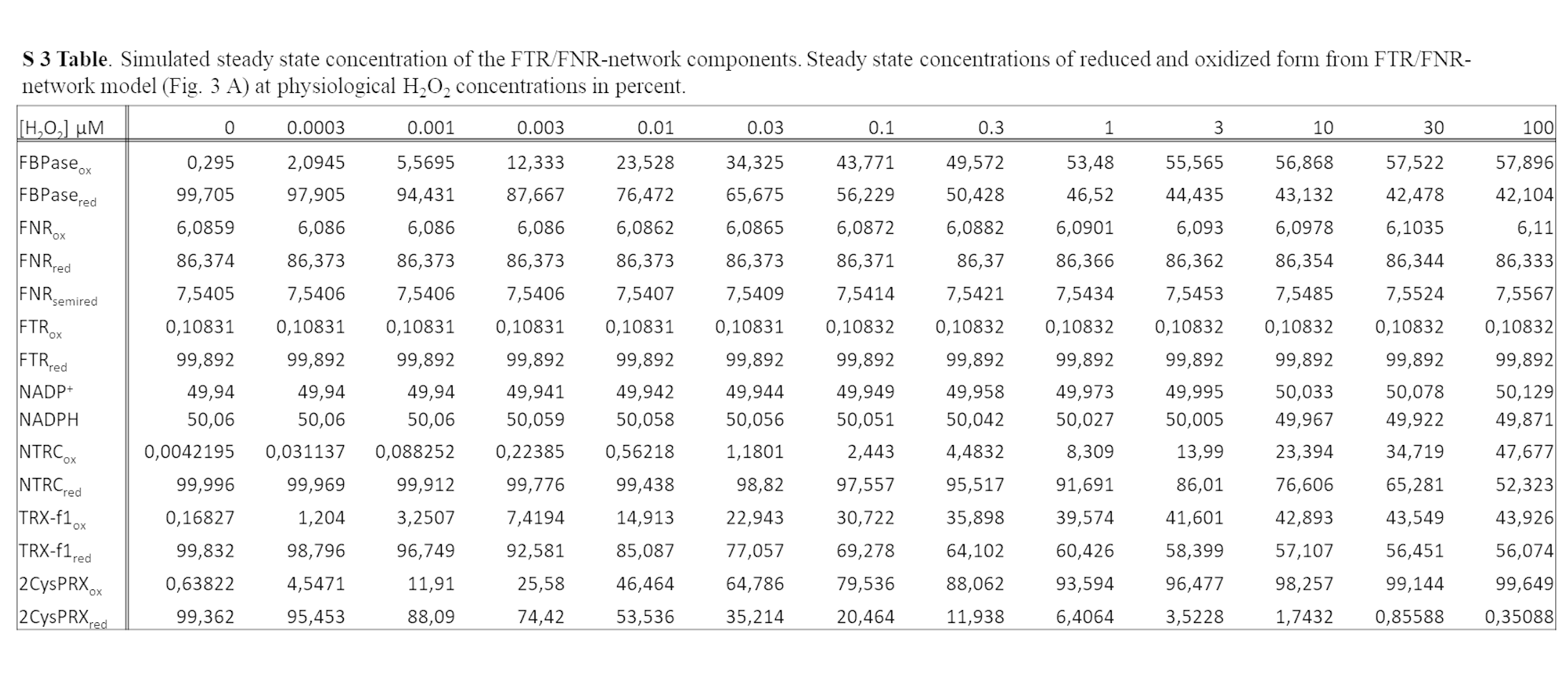

Supplement: S3 Table — (TIFF) [file pcbi.1007102.s010.tiff]

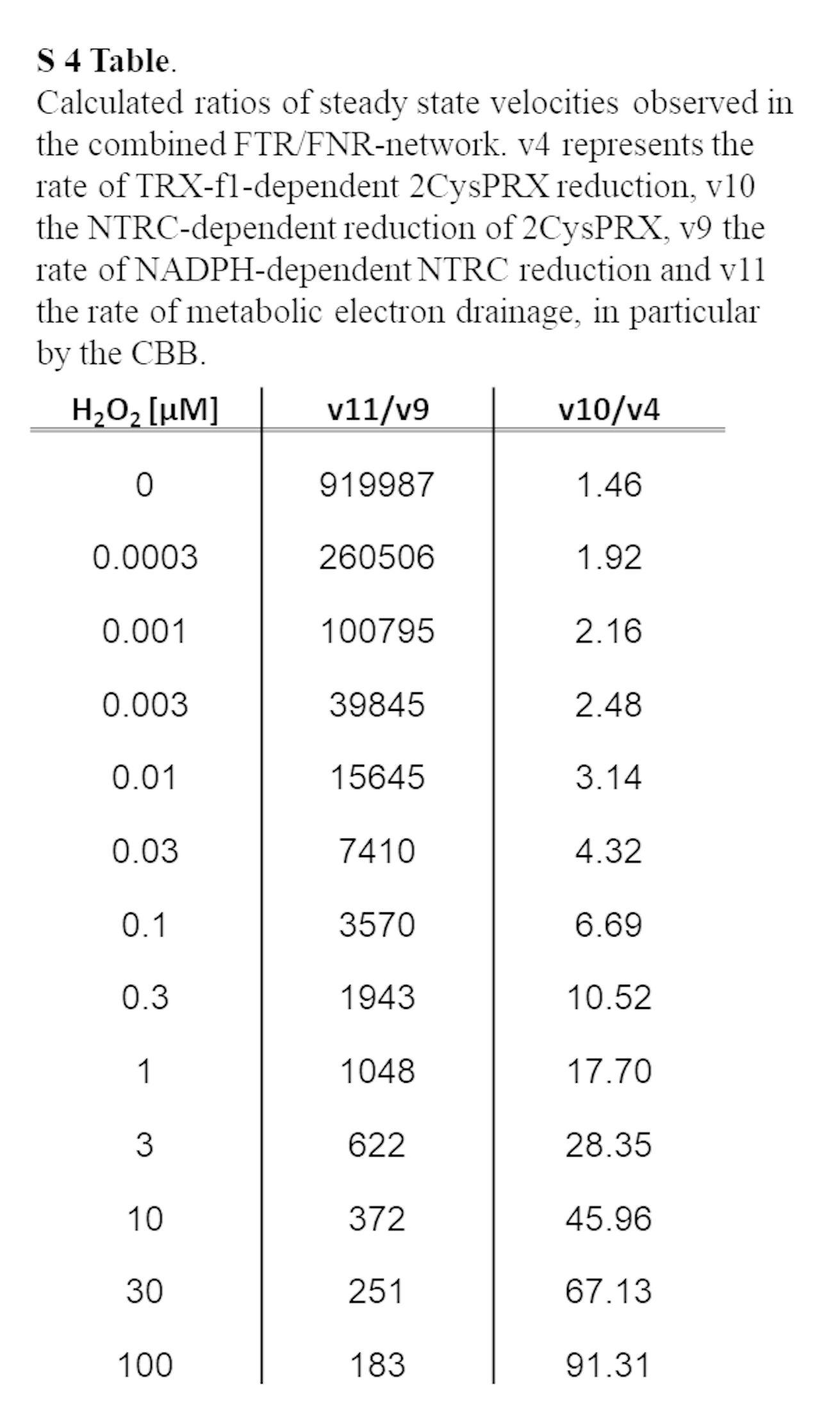

Supplement: S4 Table — (TIFF) [file pcbi.1007102.s011.tiff]

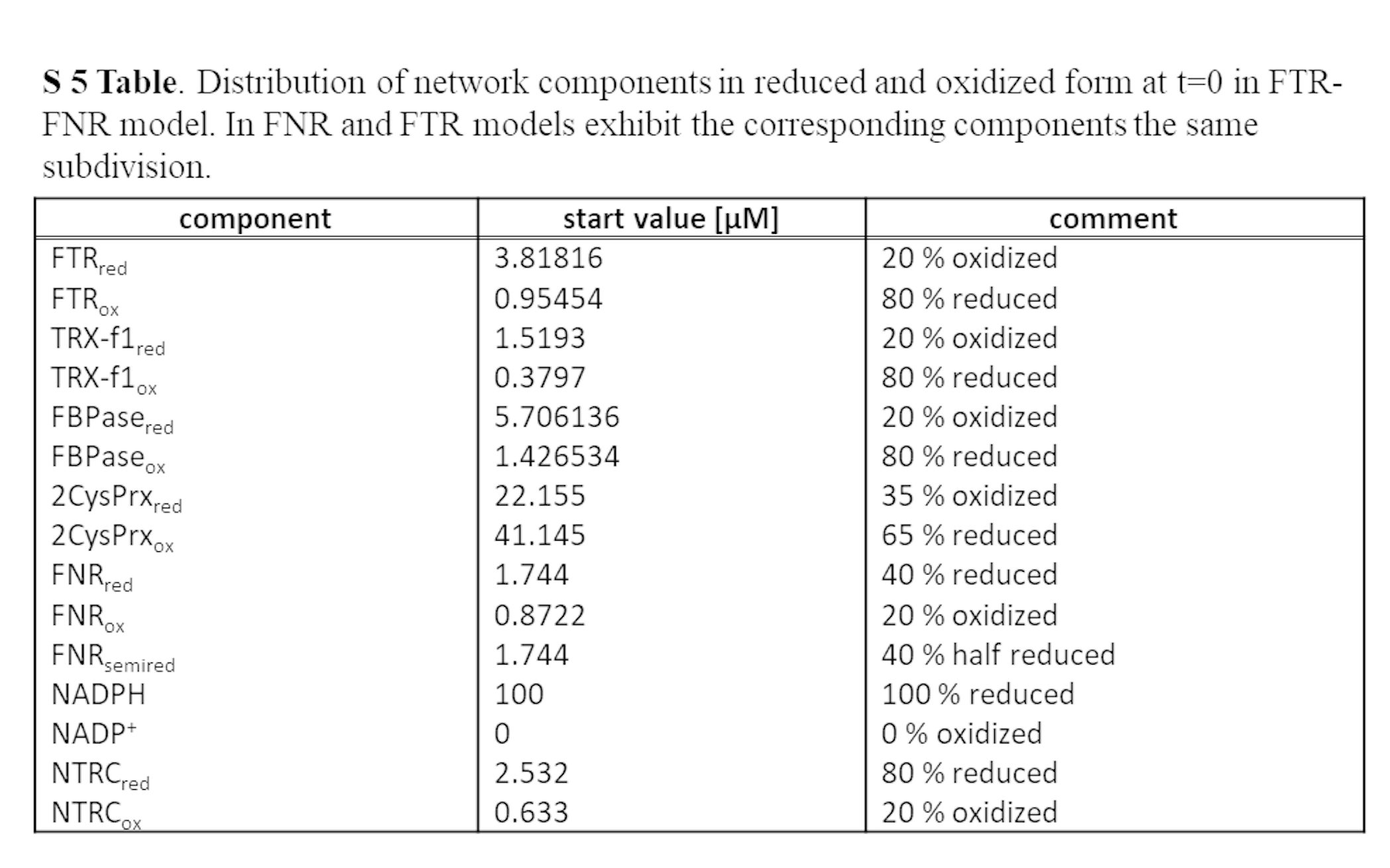

Supplement: S5 Table — (TIFF) [file pcbi.1007102.s012.tiff]

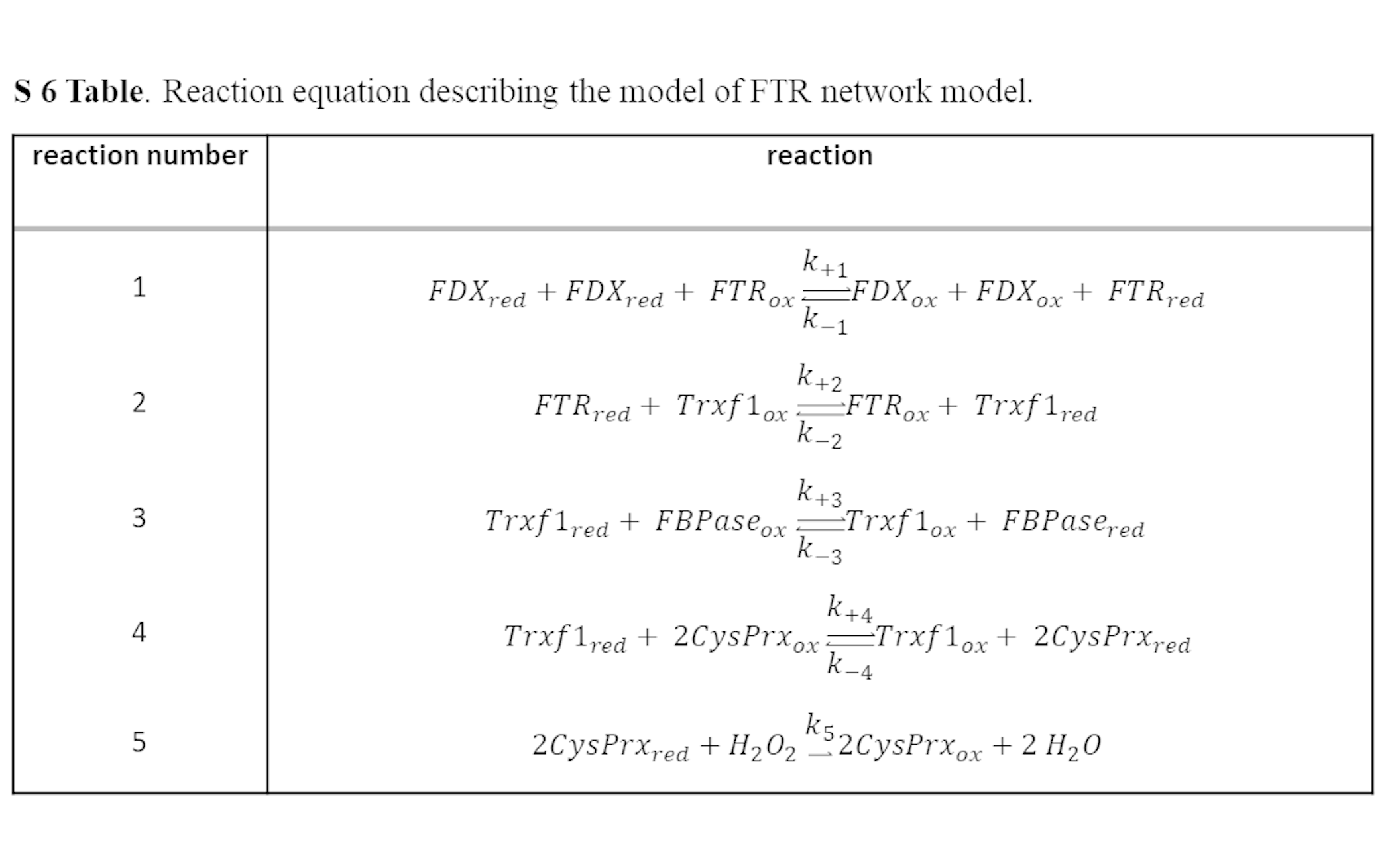

Supplement: S6 Table — (TIFF) [file pcbi.1007102.s013.tiff]

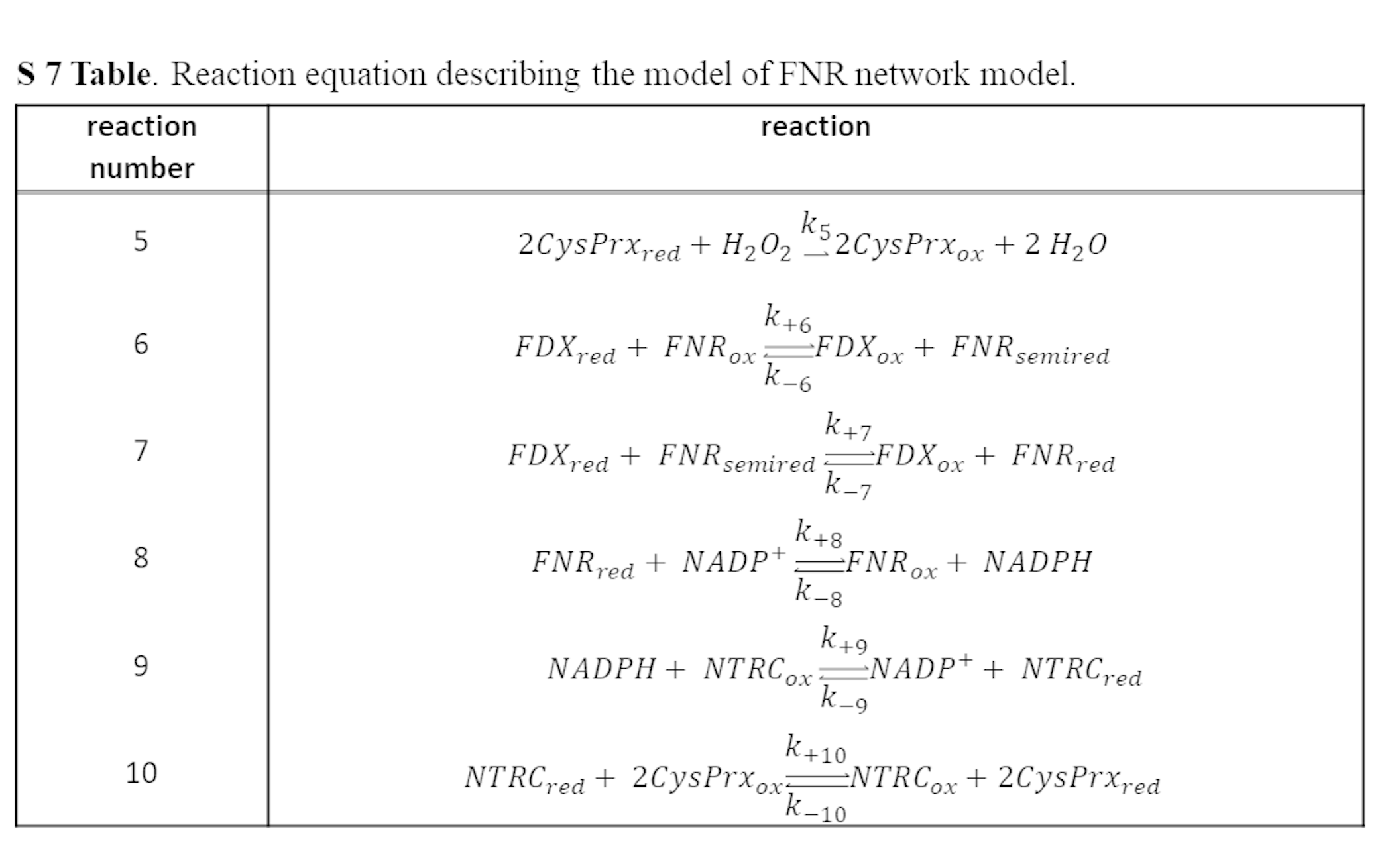

Supplement: S7 Table — (TIFF) [file pcbi.1007102.s014.tiff]

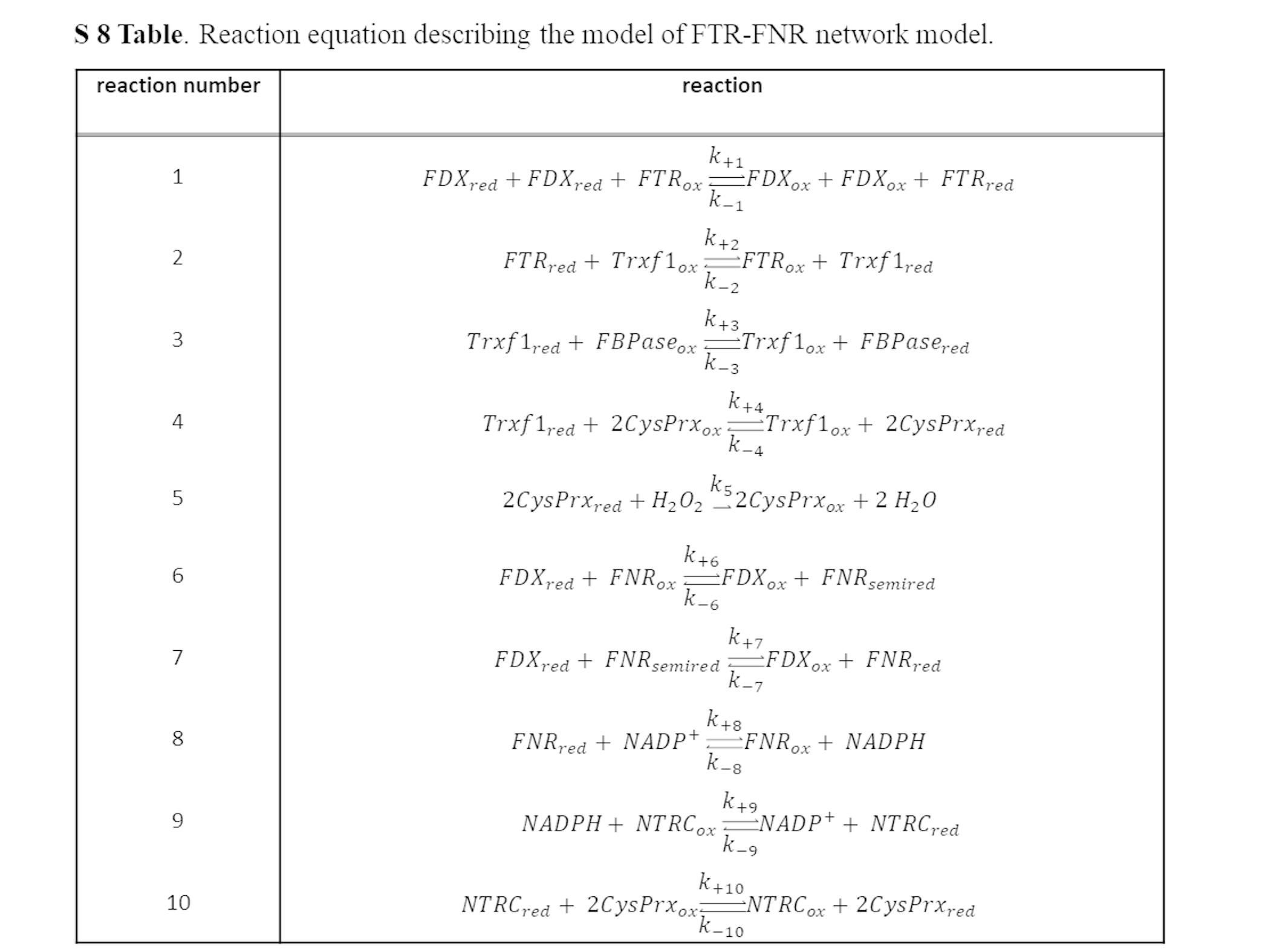

Supplement: S8 Table — (TIFF) [file pcbi.1007102.s015.tiff]
